# Temporal changes in obesity and sleep habits in Hong Kong Chinese school children: a prospective study

**DOI:** 10.1038/s41598-019-42346-z

**Published:** 2019-04-10

**Authors:** Lee-Ling Lim, Gary Tse, Kai Chow Choi, Jihui Zhang, Andrea O. Y. Luk, Elaine Chow, Ronald C. W. Ma, Michael H. M. Chan, Yun Kwok Wing, Alice P. S. Kong, Juliana C. N. Chan

**Affiliations:** 1 Department of Medicine and Therapeutics, The Chinese University of Hong Kong, Shatin, Hong Kong SAR China; 2 Asia Diabetes Foundation, Shatin, Hong Kong SAR China; 3 Department of Medicine, Faculty of Medicine, University of Malaya, Kuala Lumpur, 50603 Malaysia; 4 Li Ka Shing Institute of Health Sciences, Faculty of Medicine, The Chinese University of Hong Kong, Shatin, Hong Kong SAR China; 5 The Nethersole School of Nursing, The Chinese University of Hong Kong, Shatin, Hong Kong SAR China; 6 Department of Psychiatry, The Chinese University of Hong Kong, Shatin, Hong Kong SAR China; 7 Department of Chemical Pathology, The Chinese University of Hong Kong, Shatin, Hong Kong SAR China

## Abstract

We examined the temporal changes in obesity and sleep habits and their relationship in a prospective cohort of healthy Chinese adolescents. We collected data on anthropometric and questionnaire-measured sleep parameters in 2007–2008. 516 participants returned for examinations in 2013–2015. General obesity was defined as body mass index (BMI) ≥age- and sex-specific 95^th^ percentile or ≥25 kg/m^2^ for participants aged <18 or ≥18 years, respectively. Central obesity was defined as waist circumference (WC) ≥ age- and sex-specific 90^th^ percentile or using adult cut-offs. After a mean follow-up of 6.2 ± 0.5 years, the mean BMI increased from 18.5 ± 3.1 to 20.9 ± 3.4 kg/m^2^. The corresponding WC were 63.7 ± 8.9 and 69.8 ± 9.7 cm. General obesity rate increased from 8.3% (95% confidence interval [CI] 6.1–11.1) to 11.3% (8.7–14.4; p = 0.034). Central obesity rate decreased from 16.9% (13.7–20.4) to 13.5% (10.6–16.8; p = 0.034). During follow-up, more participants reported short sleep (<7 hours/day during weekday: 20.5% [17.1–24.2] *vs*. 15.3% [12.3–18.8]; p = 0.033) and bedtime after midnight (60.5% [56.2–64.8] *vs*. 16.2% [13.1–19.7]; p < 0.001) than baseline. The relative risk of overweight/obesity in participants with short sleep and late bedtime was 1.30 (0.48–3.47) and 1.46 (0.70–3.05), respectively. Despite rising rates of unhealthy sleep habits and general obesity, their associations were not significant at 6-year of follow-up.

## Introduction

Obesity arises as a result of a complex interplay between genetic and environmental factors including diet, physical exercise and sleep behaviour^1–3^. Technology (such as prolonged television viewing and video games) and lifestyle changes contribute to the global epidemic of obesity and sleep problems^4–6^. Although a recent systematic review of 12 studies reported a decreasing trend of mean sleep duration from the 1960s until 2000s in a few Asian and European populations^7^, there is a dearth of data exploring temporal changes in sleep duration in children and adolescents.

In a large-scale survey conducted in mainland China involving 145,078 children from 46 kindergartens, the prevalence of obesity (defined as body mass index [BMI] z-score >2 standard deviation [SD]) in those aged 5–6 years had risen from 8.8% in 2006 to 10.1% in 2010, followed by a plateau period until 2014^8^. By contrast, published data about the temporal changes in the rates of overweight and obesity among children in Hong Kong are lacking. Based on data from the Student Health Centre (SHC) of the Hong Kong’s Department of Health^9,10^, despite a progressive increase in obesity rate (defined as weight > median weight for height × 120%) since 1995, this survey had a few limitations including a lack of definition of overweight and potential overestimation of the obesity rate^11^. Apart from inattention to health check-ups due to competing priorities among school-going children, assessment at the SHC was available by appointment on a voluntary basis without any prior reminder. This might have caused selection bias as only very motivated parents or their children would return for assessment at SHC. Given a lack of prospective data on obesity and sleep habits in Hong Kong adolescents, we examined their temporal changes and relationship using a territory-wide population-based cohort established in 2007–2008, who returned for follow-ups in 2013–2015.

## Results

The baseline characteristics of the present study cohort (n = 516) are shown in Table 1. The baseline characteristics of those who did and did not participate in the follow-up study were comparable, except for a slightly lower proportion of males in the follow-up subsample. The changes in sleep duration and patterns and cardiometabolic risk factors are shown in Table 2. At baseline, 7.3% (95% confidence interval [CI] 5.2–9.9) of the cohort had a sleep duration of <6.5 hours per day during weekdays. After a mean ± SD follow-up of 6.2 ± 0.5 years, there was a trend of an increased proportion of participants with sleep duration <6.5 hours per day during weekdays [10.7% (CI 8.2–13.7)], although there was no statistically significant difference when compared to baseline (p = 0.105). By contrast, 15.3% (CI 12.3–18.8) of the cohort had short sleep using a higher cut-off of <7 hours per day, increasing significantly to 20.5% (CI 17.1–24.2) during follow-up (p = 0.033). When using weekly average values, 2.0% (CI 1.0–3.6) of the cohort had sleep duration <6.5 hours per day, rising to more than double to 5.1% (CI 3.4–7.4) during follow-up (p = 0.016). With a higher cut-off of <7 hours, 6.3% (CI 4.4–8.8) had short sleep, increasing to 11.0% (CI 8.4–14.1) during follow-up (p = 0.01). Moreover, only 16.2% (CI 13.1–19.7) of the cohort had weekly average bedtime later than midnight at baseline, but this increased by 3.7 times to 60.5% (CI 56.2–64.8; p < 0.001) during follow-up.

**Table 1:** Demographic, anthropometric and biochemical characteristics of the study cohort (n = 516).

Characteristics	Baseline	Follow-up	p-value
Male sex^a^	194 (37.6%)	194 (37.6%)	NA
Age (years)	12.8 ± 3.6	19.0 ± 3.6	**<0**.001^***^
Body weight (kg)	42.4 ± 13.4	56.2 ± 12.0	**<0**.001^***^
Body height (cm)	149.4 ± 16.0	163.3 ± 8.2	**<0**.001^***^
Body mass index (kg/m^2^)	18.5 ± 3.1	20.9 ± 3.4	**<0**.001^***^
Body mass index z-score	0.11 ± 1.09	0.16 ± 1.01	0.360
Waist circumference (cm)	63.7 ± 8.9	69.8 ± 9.7	**<0**.001^***^
Waist-to-hip ratio	0.78 ± 0.07	0.76 ± 0.06	**<0**.001^***^
Systolic blood pressure (mmHg)	108.3 ± 11.1	111.2 ± 10.0	**<0**.001^***^
Diastolic blood pressure (mmHg)	66.0 ± 8.5	70.3 ± 7.8	**<0**.001^***^
HDL–cholesterol (mmol/L)	1.63 ± 0.34	1.58 ± 0.37	**0**.004^**^
LDL–cholesterol (mmol/L)	2.21 ± 0.64	2.30 ± 0.64	**<0**.001^***^
Fasting triglyceride (mmol/L)^b^	0.70 (0.60–1.00)	0.80 (0.60–1.00)	**0**.049^*^
Fasting plasma glucose (mmol/L)	4.70 ± 0.36	4.58 ± 0.61	**<0**.001^***^

Footnotes: Data are presented as mean ± standard deviation, number (percentage)^a^ or median (interquartile range)^b^, as appropriate. The present study was based on an established cohort of 2053 Hong Kong Chinese school children and adolescents aged 6–20 years who were surveyed in 2007–2008. Using available funding support and resources, we randomly selected 580 subjects from the original cohort for a prospective follow-up between 2013 and 2015. Among them, 516 subjects (89%) agreed to participate in the follow-up study. At baseline and during follow-up, all participants had clinical and biochemical assessments including anthropometric measurements and blood tests after an overnight fast of 8–10 hours. Boldface indicates statistical significance (^*^p < 0.05, ^**^p < 0.01, ^***^p < 0.001). Fasting triglyceride was logarithmically-transformed due to skewed distribution. We used paired *t*-test for within-subject changes. HDL, high-density lipoprotein; LDL, low-density lipoprotein; NA, not applicable.

**Table 2:** Prevalence of short sleep and cardiometabolic risk factors in 516 adolescents.

	Baseline	Follow-up	p-value
Weekday sleep duration <6.5 hours per day	7.3% (5.2–9.9)	10.7% (8.2–13.7)	0.105
Weekday sleep duration <7 hours per day	15.3% (12.3–18.8)	20.5% (17.1–24.2)	**0**.033^*^
Weekly average sleep duration <6.5 hours per day	2.0% (1.0–3.6)	5.1% (3.4–7.4)	**0**.016^*^
Weekly average sleep duration <7 hours per day	6.3% (4.4–8.8)	11.0% (8.4–14.1)	**0**.010^*^
Weekly average bedtime later than midnight	16.2% (13.1–19.7)	60.5% (56.2–64.8)	**<0**.001^**^
Overweight or obesity (BMI ≥ age- and sex-specific 85^th^ percentile)^a^	20.3% (17.0–24.1)	23.4% (19.7–27.3)	0.114
General obesity (BMI ≥ age- and sex-specific 95^th^ percentile)^a^	8.3% (6.1–11.1)	11.3% (8.7–14.4)	**0**.034^*^
Central obesity (WC ≥ age- and sex-specific 90^th^ percentile)	16.9% (13.7–20.4)	13.5% (10.6–16.8)	**0**.034^*^
High triglyceride level	4.8% (3.1–7.0)	4.8% (3.1–7.0)	0.999
Low HDL-cholesterol level	3.0% (1.7–4.9)	4.8% (3.1–7.0)	0.200
High blood pressure level	3.5% (2.1–5.5)	5.1% (3.4–7.5)	0.185
High fasting plasma glucose level	2.0% (1.0–3.6)	0.6% (0.1–1.7)	0.109
Metabolic syndrome^b^	0.6% (0.1–1.7)	1.8% (0.8–3.4)	0.146

Footnotes: Data are presented as prevalence (95% confidence interval). Boldface indicates statistical significance (^*^p < 0.05, ^**^p < 0.001). We used McNemar’s test for within-subject changes. ^Ψ^Overweight or obesity was defined by Cook’s criteria with percentile curves based on a local population survey. ^a^Obesity was defined as body mass index (BMI) ≥ age- and sex-specific 95^th^ percentile or ≥25 kg/m^2^ for those aged <18 or ≥18 years, respectively. The corresponding cut-offs for overweight were BMI ≥ age- and sex-specific 85^th^ percentile or ≥23 kg/m^2^. ^b^Metabolic syndrome was defined by the International Diabetes Federation criteria: central obesity (waist circumference [WC] ≥ age- and sex-specific 90^th^ percentile or adult cut-offs) plus ≥2 of the following: triglyceride ≥1.7 mmol/L, high-density lipoprotein cholesterol (HDL-cholesterol) < 1.03 mmol/L, blood pressure ≥130/85 mmHg, fasting plasma glucose ≥5.6 mmol/L (adult criteria were adopted for adolescents aged 16 years or older).

In terms of cardiometabolic risk factors, 20.3% (CI 17.0–24.1) were either overweight or obese using a BMI ≥ age- and sex-specific 85^th^ percentile, rising to 23.4% (CI 19.7–27.3) during follow-up (p < 0.001). For general obesity (BMI ≥ age- and sex-specific 95^th^ percentile), the proportion increased from 8.3% (CI 6.1–11.1) to 11.3% (CI 8.7–14.4) (p = 0.034). Opposite to general obesity which was measured by BMI, there was a significant reduction of central obesity (WC ≥ age- and sex-specific 90^th^ percentile) from 16.9% (CI 13.7–20.4) at baseline to 13.5% (CI 10.6–16.8) during follow-up (p = 0.034). For high triglyceride, low HDL-cholesterol, high blood pressure, high fasting plasma glucose or metabolic syndrome, there was no significant temporal change in the proportion of participants affected (all p > 0.05).

We examined the relationship between short sleep and late bedtime on the risk of developing obesity amongst normal weight adolescents at baseline (n = 411) (Table 3). Compared to adolescents with weekly average sleep duration ≥7 hours, those with <7 hours of sleep did not have a significant increase in risk of developing either overweight or obesity during follow-up, even after adjusting for age and sex (unadjusted relative risk [RR]: 1.40 [CI 0.54–3.67] and adjusted RR: 1.30 [CI 0.48–3.47]). Similarly, there was no significant increase in risk of obesity alone (unadjusted RR: 1.18 [CI 0.29–4.76] and adjusted RR: 1.27 [CI 0.29–5.50]). Comparisons were made between adolescents with weekly average bedtime after midnight to those with weekly average bedtime before midnight for overweight or obesity, but no significant increase in risk was observed (unadjusted RR: 1.48 [CI 0.76–2.88] and adjusted RR: 1.46 [0.70–3.05]). Again, for obesity alone, the risk was not statistically significant (unadjusted RR: 0.91 [CI 0.32–2.56] and adjusted RR: 0.93 [CI 0.30–2.87]).

**Table 3:** The relationships of short sleep and late bedtime with risk of developing overweight or obesity amongst adolescents with normal weight at baseline (n = 411).

	Overweight or obesity during follow-up		Obesity during follow-up	
**Baseline sleep duration**				
Weekly average sleep duration ≥7 hours	9.8% (7.0–13.4)		5.6% (3.7–8.3)	
Weekly average sleep duration <7 hours	13.8% (3.9–31.7)		6.7% (0.8–22.1)	
Unadjusted RR (95% CI)	1.40 (0.54–3.67)	p = 0.491	1.18 (0.29–4.76)	p = 0.815
Age- and sex-adjusted RR (95% CI)	1.30 (0.48–3.47)	p = 0.608	1.27 (0.29–5.50)	p = 0.748
**Baseline bedtime**				
Weekly average bedtime ≤0:00 (midnight)	9.3% (6.4–13.0)		5.8% (3.7–8.6)	
Weekly average bedtime >0:00 (midnight)	13.7% (6.8–23.8)		5.3% (1.5–12.9)	
Unadjusted RR (95% CI)	1.48 (0.76–2.88)	p = 0.255	0.91 (0.32–2.56)	p = 0.857
Age- and sex-adjusted RR (95% CI)	1.46 (0.70–3.05)	p = 0.310	0.93 (0.30–2.87)	p = 0.901

Footnotes: Data are presented as prevalence (%) or relative risk (RR) with 95% confidence interval (CI). Relative risk was defined as risk of developing overweight or obesity during follow-up in adolescents with short sleep (or late bedtime), compared to those without a history of short sleep (or late bedtime).

## Discussion

From this prospective cohort study in Hong Kong Chinese adolescents, we reported a significant increase in general obesity rate from 8.3% to 11.3%, but a decrease in central obesity rate from 16.9% to 13.5%. We also reported an increased proportion of adolescents reporting short sleep duration (defined as <7 hours per day during weekdays) from 15.3% to 20.5% and late bedtime after midnight from 16.2% to 60.5%. Despite the similar rising trends, the associations between unhealthy sleep habits and obesity were not statistically significant at 6-year of follow-up.

Globally, a total of 107.7 million children aged <20 years were obese in 2015^4^. Of note, the prevalence of overweight and obesity during adolescence and young adulthood has doubled in the last three decades, with its rate of increase greater than adult obesity in most countries^4^. This is an important problem that needs tackling because childhood obesity is a strong predictor for cardiometabolic diseases in subsequent years of life^12–14^. Apart from slow progress in health policy targeting food environments and health systems reforms (such as personal healthcare access and quality)^15^, other environmental risk factors such as abnormal sleep duration and patterns may contribute to the obesity epidemic^6^. In this regard, a meta-analysis of randomized controlled trials showed that short sleep could possibly increase food intake and body weight, whilst sleep improvement interventions were associated with weight loss in children and adult populations^16^. Another meta-analysis of 17 observational studies found that children with short sleep duration had 58–92% higher odds for overweight or obesity than those with longer sleep duration^17^. Although an updated systematic review of 15 studies found a significant negative relationship between sleep duration and obesity risk^18^, the majority of included studies were cross-sectional in design and therefore, a causal relationship could not be established.

By using a prospective cohort with a mean follow-up of six years, we observed an increased rate of general obesity but not for central obesity, along with an increased proportion of adolescents with abnormal sleep habits (either short sleep or late bedtime after midnight). In adults, abnormal sleep habits contribute to an excess risk of obesity, driven by alterations in eating behaviour, satiety hormones (reduced leptin to ghrelin ratio), autonomic nervous systems and hypothalamic-pituitary-adrenal axis^6^. During puberty, the pathogenesis of obesity is even more complex with additional influences from sex steroids and sex steroids-stimulated growth hormone (GH) release^19^. Compared to boys, several studies reported that girls were more likely to have subcutaneous fat than visceral fat accumulation especially during late puberty or early adulthood^20,21^. This could be attributable to an increase in oestradiol levels, as well as site- and sex-specific distributions of receptors and the enzymatic activity of sex steroids within the fat depots^19,22,23^. Hence, the presence of a high proportion of girls (~62%) in our cohort could possibly explain the increasing rate of general obesity but not central obesity. In addition, high testosterone (which was then aromatized to oestradiol) and sex steroids-induced GH levels in boys were shown to upregulate GH/catecholamines-sensitive beta-adrenergic receptors and inhibit lipoprotein lipase activity in adipocytes, which enhanced lipolysis and the redistribution of visceral adipose tissue to subcutaneous depots during puberty^19,23^. Chronic short sleep was also associated with prolonged exposure to increased GH levels as sequelae of a biphasic nocturnal GH pulse^6^. Of note, existing evidence on the temporal onset of sex differences in fat distribution has been inconsistent, perhaps due to variations in ethnicity, methods of anthropometric measurements, the timing of puberty and hormonal status^20,24,25^. Taken together, these findings favoured an increasing BMI amongst adolescents with short sleep duration.

Although the age- and sex-adjusted relative risks of overweight and/or obesity were all greater than unity for short sleep duration, no statistical significance was reached. This may be due to the relatively small sample size and short duration of follow-up of our cohort which limits the statistical power of the present analysis to elucidate the relationships between sleep habits and obesity. In a prospective cohort study involving 597 pre-pubertal children conducted in Canada, after three to eight years of follow-up, there was also no significant association between sleep duration (cross-sectional assessment during the age of 1–3 years) and BMI^26^. On the other hand, a meta-analysis of three prospective observational studies with longer than one-year follow-up found that short sleep duration was associated with a 30% higher risk of overweight or obesity in adolescents^27^. In a large prospective cohort study involving >23,000 students aged 7–18 years from mainland China, short sleep was significantly associated with the development of obesity at a later stage^28^. Apart from nearly half of the cohort were children aged 7–12 years, more than one-third of these children and adolescents reported short sleep (defined as <7 hours per day), which was higher than the present study (15.3–20.5%)^28^. Taken together, the prevalence of short sleep or late bedtime, the magnitude of short sleep duration and the potential confounding elements of sleep compensation and sleep quality, might all play roles in modulating the development of obesity amongst children and adolescents. More prospective studies that address these factors will provide additional insights into this increasingly important relationship.

To our knowledge, this was the first prospective study which examined the relationship between abnormal sleep habits and risk of obesity in Hong Kong Chinese adolescents. We performed a longitudinal assessment of sleep patterns which helped to reduce recall bias. We also excluded adolescents with a known history of endocrine disorders, growth problem or obstructive sleep apnoea.

Several limitations should be acknowledged. First, given the sexual dimorphism in fat distribution, a larger cohort would allow differentiation of association by gender. However, due to limited funding and resources, only about one-quarter of the original cohort had a prospective follow-up. Second, apart from the adjustment of age and sex in the association analysis, we were not able to consider the confounding effects of other lifestyle, behavioural and socio-economic factors due to limitations in the data source. Third, there was a lack of objective assessment of pubertal timing and stages, albeit our cohort was likely to be in the early- to middle pubertal stage as the mean age of pubertal onset ranged from 9.4 to 11.7 years in Hong Kong children^29^. Fourth, additional hormonal assessment including sex steroids, IGF-1 and hormones modulating satiety such as leptin and ghrelin, may further explain the differences in obesity rates with abnormal sleep habits. Fifth, although we did not use either magnetic resonance imaging or dual-energy X-ray absorptiometry for the assessment of fat distribution, waist circumference has been recognized as a reliable, cost-effective and commonly adopted field technique for determining central obesity^30^. Last, we used a sleep-reported sleep questionnaire and did not capture information about sleep quality. Although objective sleep measurements such as actigraphy and polysomnography are the gold standards for assessing sleep duration, patterns and quality, these are labour intensive and not practically feasible for large-scale screening. Nevertheless, our prior work had indicated the reliability of the locally-validated sleep questionnaire with good correlation with actigraphy^3,31^.

While both rates of general obesity and unhealthy sleep habits increased in Hong Kong adolescents, their associations were not significant after six years of follow-up. Nevertheless, our observation that more than half of the present cohort reported late bedtime after midnight is alarming, calling for public health attention. Larger prospective studies allowing subgroup analyses for gender differences and categorization according to age groups and pubertal stages are needed to validate these observations, as well as to elucidate the effects of poor sleep quality and their interactions with other environmental, molecular and genetic factors, in order to facilitate obesity prevention in the youth populations.

## Methods

### Study populations

The present study was based on an established cohort of 2053 Hong Kong Chinese school children and adolescents aged 6–20 years who were surveyed in 2007–2008^3^. Using available funding support and resources, we randomly selected 580 subjects from the original cohort for a prospective follow-up between 2013 and 2015. Among them, 516 subjects (89%) agreed to participate in the follow-up study. The remaining 64 subjects (11%) either declined to participate or were not contactable due to a change to the contact number or home address. At baseline and during follow-up, after an overnight fast of 8–10 hours, all participants had comprehensive clinical and biochemical assessment including anthropometric measurements and blood tests (plasma glucose, lipid profile, complete blood count, renal and liver function tests).

Sleep habits were assessed using a validated questionnaire (Supplementary Notes S1 and S2)^31^, which showed a good agreement with actigraphy-measured sleep duration with an intraclass correlation coefficient of 0.72^3^. Short sleep duration was defined as <6.5 hours per day derived from our prior work^3,31^ or <7 hours per day as recommended by National Sleep Foundation^32^. We estimated the weekly average sleep duration using a weighted average: (5 × weekdays sleep duration + 2 × weekend sleep duration)/7. We used a similar equation for estimating the weekly average bedtime. General obesity was defined as body mass index (BMI) ≥ age- and sex-specific 95^th^ percentile or ≥25 kg/m^2^ for participants aged < 18 or ≥18 years, respectively^33–35^. Central obesity was defined as ≥ age- and sex-specific 90^th^ percentile of WC or adult cut-offs^36,37^. The BMI and WC cut-off values used in the present study were based on local population-based age- and sex-specific percentiles^35,37^. We defined metabolic syndrome using the International Diabetes Federation criteria, namely the presence of central obesity plus at least two of the following criteria: triglyceride ≥1.7 mmol/L, high-density lipoprotein cholesterol (HDL-cholesterol) <1.03 mmol/L, blood pressure ≥130/85 mmHg, fasting plasma glucose ≥5.6 mmol/L (adult criteria were adopted for adolescents aged 16 years or older)^38^. This study was approved by the Chinese University of Hong Kong-New Territories East Cluster Clinical Research Ethics Committee. Written informed consents were obtained from either a parent or a legal guardian. All methods were performed in accordance with the relevant guidelines and regulations.

### Laboratory assays

Fasting plasma glucose (hexokinase method) and lipid profile including total cholesterol (enzymatic method), triglyceride (enzymatic method without glycerol blanking) and HDL-cholesterol, direct method using PEG-modified enzymes and dextran sulfate) were measured on a Roche Modular Analytics system (Roche Diagnostics GmbH, Mannheim, Germany) using standard reagent kits supplied by the manufacturer. All laboratory assays were performed using standard methods by the Department of Chemical Pathology at the Prince of Wales Hospital.

### Statistical analysis

Data were presented using appropriate descriptive statistics. Normality of continuous variables was assessed on the basis of their skewness statistics and normal probability plots. Triglyceride and urinary albumin:creatinine ratio (ACR) were logarithmically-transformed before entering them into the inferential analysis. We presented the prevalence of unhealthy sleep habits, obesity and other cardiometabolic risk factors in percentages with 95% CI and compared their values at baseline and during follow-up using McNemar’s test. We used paired *t*-test to assess the changes in participants’ demographic, anthropometric and biochemical characteristics at baseline and during follow-up. After excluding 105 adolescents with either overweight or obesity at baseline, we used log-binomial regression model to assess the RR of developing overweight or obesity during follow-up in normal weight adolescents with either short sleep duration (defined as <6.5 hours or <7 hours per day) or bedtime later than midnight at baseline. All statistical analyses were performed using SAS (SAS Institute, Cary, NC, release 9.3). A two-sided p-value < 0.05 was considered significant.

## Supplementary information

Supplementary Notes S1 and S2

## Data Availability

Data will be made available upon reasonable request.
