# Supplementary material for: Temporal changes in obesity and sleep habits in Hong Kong Chinese school children: a prospective study
Source: Sci Rep. 2019 Apr 10;9:5881. doi: 10.1038/s41598-019-42346-z (PMC6458117; doi:10.1038/s41598-019-42346-z)
Supplement: Supplementary file 1 — Supplementary Notes S1 and S2 [file 41598_2019_42346_MOESM1_ESM.docx]

**Temporal changes in obesity and sleep habits in Hong Kong Chinese school children: a prospective study (Supplementary materials)**

Lee-Ling Lim^1,2,3†^, Gary Tse^1,4†^, Kai Chow Choi^5^, Jihui Zhang^6^, Andrea O.Y. Luk^1,2,4^, Elaine Chow^1^, Ronald C.W. Ma^1,4^, Michael H.M. Chan^7^, Yun Kwok Wing^6^, Alice P.S. Kong^1,4*^, Juliana C.N. Chan^1,2,4^

^1^Department of Medicine and Therapeutics, The Chinese University of Hong Kong, Shatin, Hong Kong SAR, China

^2^Asia Diabetes Foundation, Shatin, Hong Kong SAR, China

^3^Department of Medicine, Faculty of Medicine, University of Malaya, Kuala Lumpur, 50603, Malaysia

^4^Li Ka Shing Institute of Health Sciences, Faculty of Medicine, The Chinese University of Hong Kong, Shatin, Hong Kong SAR, China

^5^The Nethersole School of Nursing, The Chinese University of Hong Kong, Shatin, Hong Kong SAR, China

^6^Department of Psychiatry, The Chinese University of Hong Kong, Shatin, Hong Kong SAR, China

^7^Department of Chemical Pathology, The Chinese University of Hong Kong, Shatin, Hong Kong SAR, China

^*^Correspondence:

Dr. Alice PS Kong,

Department of Medicine and Therapeutics,

Prince of Wales Hospital, The Chinese University of Hong Kong,

Shatin, N.T., Hong Kong SAR, China.

Phone: (852) 3505 1558; Fax: (852) 2637 3852

E-mail address: [alicekong@cuhk.edu.hk](mailto:alicekong@cuhk.edu.hk)

**Supplementary Note S1. Sleep habits questionnaire (English version)**

In the past one month,

1. Do you need to do shift work?

0□ Not at all 1□ Used to, but have quit more than 1 year

2□ Sometimes 3□ Frequent

1. What time do you usually go to bed and wake up in the morning?

|  | Bedtime | Wake time |
| --- | --- | --- |
| 1. Sunday to Thursday |  |  |
| 1. Friday, Saturday, or rest day |  |  |

1. How long (in minutes) do you need to fall asleep each night?

1□ less than 15 minutes 2□ 16-30 minutes 3□ 31-60 minutes

4□ 61-120 minutes 5□ more than 120 minutes (about ____ minutes)

1. a. Do you think you have enough sleep? 0□ not enough 1□ enough

b. How long do you think it would be enough for you? ______hour(s)_____minute(s)

1. a. Do you have the habit of daytime napping?

0□ Not at all 1□ would like to, but cannot fall asleep 2□ 1-2 days per week

3□ 3-5 days per week 4□ every day or almost every day 5□ not sure

b. How long (in minutes) did you sleep? ________minutes

How likely are you to doze off or fall asleep in the following situations?

0 = would never doze

1 = slight chance of dozing

2 = moderate chance of dozing

3 = high chance of dozing

| 6. Watching TV | 0 | 1 | 2 | 3 |
| --- | --- | --- | --- | --- |
| 7. Sitting and reading | 0 | 1 | 2 | 3 |
| 8. Sitting, inactive in a public place (e.g. a theatre or a meeting) | 0 | 1 | 2 | 3 |
| 9. As a passenger in a car for an hour without a break | 0 | 1 | 2 | 3 |
| 10. Lying down to rest in the afternoon when circumstances permit | 0 | 1 | 2 | 3 |
| 11. Sitting and talking to someone | 0 | 1 | 2 | 3 |
| 12. Sitting quietly after lunch without alcohol | 0 | 1 | 2 | 3 |
| 13. In a car, while stopped for a few minutes in the traffic | 0 | 1 | 2 | 3 |

During the past month, how often have you had trouble sleeping because you…

|  | Not during the past month (0) | Less than once a month (1) | Once or twice a month (2) | Once or twice a week (3) | Three or more times a week (4) |
| --- | --- | --- | --- | --- | --- |
| 14. Cannot get to sleep within 30 minutes |  |  |  |  |  |
| 15. Wake up in the middle of the night or early morning |  |  |  |  |  |
| 16. Wake up early in the morning (before dawn), and cannot go back to sleep |  |  |  |  |  |
| 17. Snoring during sleep |  |  |  |  |  |
| 18. Breathe with mouth during sleep |  |  |  |  |  |
| 19. Sweating during sleep |  |  |  |  |  |
| 20. Unwilling to wake up during morning |  |  |  |  |  |
| 21. Feeling nonrestorative after waking up |  |  |  |  |  |
| 22. Feeling mouth dry after waking up |  |  |  |  |  |
| 23. Feeling headache after waking up |  |  |  |  |  |
| 24. Feeling tired during the day |  |  |  |  |  |

**Supplementary Note S2. Sleep habits questionnaire (Chinese version)**

*The sleep habits questionnaire has been validated in the Hong Kong Chinese population^1^, with a good agreement with actigraphy-measured sleep duration^2^.*

**有關你最近一月的睡眠習慣**

1. 你是否需要輪班工作?

| 0 □ 不需要 | 1 □ 以前有，但最近一年沒有 | 2 □ 有時 | 3 □ 定期 |
| --- | --- | --- | --- |

2. 你通常甚麼時候**上床**睡覺，甚麼時候**起床**？

|  |  | 上床睡覺 | 起床 |
| --- | --- | --- | --- |
| a. | **星期日至四** | ______時:_____分 | ______時:_____分 |
| b. | **星期五、六或第二天不用上班** | ______時:_____分 | ______時:_____分 |

3. 每晚你通常**需要多少時間才能入睡**?

| 1 □ 15分鐘或以下 | 2 □ 16-30分鐘 | 3 □ 31-60分鐘 |
| --- | --- | --- |
| 4 □ 61-120分鐘 | 5 □ 超過120分鐘 (約_______分鐘) | |

4. a 你認為你**得到足夠的睡眠**嗎? 0□ 不足夠 1□ 足夠

b. 你覺得每晚需要**睡多久才足夠**? _____ 小時______分鐘

5. a. **白天裡**，你有沒有**小睡（瞓晏覺／午睡）**的習慣呢?

| 0 □ 不需要 (跳去第5題) | 1 □ 想，但睡不著 　(跳去第5題) | 2 □ 每星期有一至兩天 |  |  |
| --- | --- | --- | --- | --- |
| 3 □ 每星期有三至五天 | 4 □ 每天或差不多每天都有 | 5 □ 不清楚 (跳去第5題) |  |  |
| b. 通常會睡多少時間? 大約___________ 分鐘 | | | |  |

| **請回答以下問題，看看閣下在日間是否容易瞌睡（眼瞓）** | 從未 | 少有 | 多數時候 | 經常 |
| --- | --- | --- | --- | --- |
| 1. 在看電視時，便打瞌睡 | 0□ | 1□ | 2□ | 3□ |
| 1. 坐下閱讀時，便打瞌睡 | 0□ | 1□ | 2□ | 3□ |
| 1. 在公眾地方安靜坐下，便打瞌睡 | 0□ | 1□ | 2□ | 3□ |
| 1. 乘坐在汽車上，不停地行駛多過一小時，便打瞌睡 | 0□ | 1□ | 2□ | 3□ |
| 1. 在中午時舒服地坐下休息時，便打瞌睡 | 0□ | 1□ | 2□ | 3□ |
| 1. 坐下與別人閒談時，便打瞌睡 | 0□ | 1□ | 2□ | 3□ |
| 1. 午飯後（並沒有渴過酒），安靜地坐下時，便打瞌睡 | 0□ | 1□ | 2□ | 3□ |
| 1. 坐在車上，當車子停在紅燈前或在塞車時，幾分鐘便打瞌睡 | 0□ | 1□ | 2□ | 3□ |

以下是一系列有關你在**過往一月內**的睡眠情況, 請選擇下列每一項描述所發生的次數**:**

|  | **從不** | **每月**  **少於一次** | **每月**  **一至二次** | **每星期**  **一至兩次** | **每星期**  **三次或以上** |
| --- | --- | --- | --- | --- | --- |
| 1. 晚上**難以入睡** | 0□ | 1□ | 2□ | 3□ | 4□ |
| 1. 睡至半夜會“**扎**”醒 | 0□ | 1□ | 2□ | 3□ | 4□ |
| 1. 早上太早醒來 **(如天沒亮)**, **便不能再入睡** | 0□ | 1□ | 2□ | 3□ | 4□ |
| 1. 睡覺時**打鼻鼾** | 0□ | 1□ | 2□ | 3□ | 4□ |
| 1. 睡覺時**用口呼吸** | 0□ | 1□ | 2□ | 3□ | 4□ |
| 1. 睡覺時**經常流汗** | 0□ | 1□ | 2□ | 3□ | 4□ |
| 1. 早上**很不願意起床** | 0□ | 1□ | 2□ | 3□ | 4□ |
| 1. 早上起床後, 覺得**好像沒有休息過** | 0□ | 1□ | 2□ | 3□ | 4□ |
| 1. 早上起床後, 覺得**口乾** | 0□ | 1□ | 2□ | 3□ | 4□ |
| 1. 早上起床後, 覺得**頭痛** | 0□ | 1□ | 2□ | 3□ | 4□ |
| 1. 白天裡, 覺得**好疲倦** | 0□ | 1□ | 2□ | 3□ | 4□ |

**References**

1 Li, A. M. *et al.* Validation of a questionnaire instrument for prediction of obstructive sleep apnea in Hong Kong Chinese children. *Pediatr Pulmonol.* 41, 1153-1160 (2006).

2 Kong, A. P. *et al.* Associations of sleep duration with obesity and serum lipid profile in children and adolescents. *Sleep Med.* 12, 659-665 (2011).
